# Advanced ALK–EML4 fusion–positive non-small cell lung cancer with intraspinal metastases: a case report

**DOI:** 10.3389/fonc.2026.1916641

**Published:** 2026-09-03

**Authors:** Juan Lang, Baoyu Guo, Qi Han, Zhongkui Xiong

**Affiliations:** 1 Department of Pathology, Shaoxing People’s Hospital, Shaoxing, Zhejiang, China; 2 School of Medicine, Shaoxing University, Shaoxing, Zhejiang, China; 3 Department of Radiation Oncology, Shaoxing Second Hospital, Shaoxing, Zhejiang, China; 4 Department of General Practice, Tashan Community Health Service Center of Yuecheng District, Shaoxing, Zhejiang, China

**Keywords:** ALK (anaplastic lymphoma kinase), anlotinib, intraspinal metastases, lorlatinib, non-small cell lung cancer, radiotherapy, spinal cord compression, targeted therapy

## Abstract

Among non-small cell lung cancer (NSCLC) cases, anaplastic lymphoma kinase (ALK)-positive disease is characterized by a chromosomal rearrangement that generates an oncogenic ALK fusion gene, occurring in approximately 2–11% of patients. ALK-positive NSCLC is associated with a high incidence of brain metastases, with at least 20% of patients presenting with brain metastases at the time of diagnosis. Conversely, intraspinal metastasis is an uncommon site of metastatic involvement in patients with NSCLC. When intraspinal metastases invade the lumbar spinal canal, they may cause low back pain as well as neurological symptoms and signs in the lower limbs. Herein, we report a case of intraspinal metastasis in a patient with ALK–EML4 fusion–positive lung adenocarcinoma. Notably, at the time of initial relapse, molecular profiling failed to detect any mutations in the epidermal growth factor receptor (EGFR) or ALK genes. However, next-generation sequencing performed at the third recurrence confirmed the presence of the ALK–EML4 fusion mutation. Following the fifth disease recurrence, the patient received palliative radiotherapy followed by combination targeted therapy with lorlatinib and anlotinib, and subsequently transitioned to maintenance therapy with lorlatinib, achieving an overall survival of 121 months (right-censored, t+) and a duration of treatment of 48 months (t+) with lorlatinib as fifth-line therapy.

## 1 Introduction

According to Cancer Statistics, 2026, lung cancer is the second most commonly diagnosed malignancy in both men and women and remains the leading cause of cancer-related death worldwide (1). According to a 2022 report, lung cancer ranks first in cancer incidence and mortality among both males and females combined in China (2). Non-small cell lung cancer (NSCLC) accounts for approximately 85% of all lung cancer cases. Among NSCLC subtypes, anaplastic lymphoma kinase (ALK)-positive disease is characterized by a chromosomal rearrangement that generates an oncogenic ALK fusion gene, occurring in approximately 2–11% of NSCLC cases (3). Crizotinib, alectinib, and lorlatinib are first-, second-, and third-generation anaplastic lymphoma kinase tyrosine kinase inhibitors (ALK-TKIs), respectively, and are clinically used for the targeted treatment of ALK-positive NSCLC.

In the open-label, phase III PROFILE 1014 trial comparing crizotinib with pemetrexed plus cisplatin/carboplatin in treatment-naïve patients with advanced ALK-positive non-squamous NSCLC, median progression-free survival (mPFS) was significantly longer in the crizotinib group than in the chemotherapy group (10.9 vs. 7.0 months), while the 1-year survival rates were 84% and 79%, respectively (4). These findings demonstrated that crizotinib is an effective first-line therapeutic option and is superior to chemotherapy in patients with ALK-positive NSCLC. Meanwhile, in the randomized, multicenter, open-label phase III ALUR study comparing alectinib with chemotherapy in patients with ALK-positive advanced or metastatic NSCLC who had previously received platinum-based chemotherapy and crizotinib, alectinib was associated with significantly longer mPFS than chemotherapy (7.1 vs. 1.6 months). In addition, the central nervous system objective response rate (ORR) was significantly higher in the alectinib group (54.2% vs. 0%) (5). This demonstrated that alectinib is a highly effective and preferred second-line therapeutic option for patients with ALK-positive NSCLC. Furthermore, updated long-term data from the CROWN trial demonstrated the durable clinical benefit of lorlatinib compared with crizotinib in treatment-naïve patients with ALK-positive NSCLC and supported the use of lorlatinib as a first-line therapy regardless of baseline brain metastasis status (6). After 7 years of follow-up, mPFS was not reached with lorlatinib, representing the longest PFS ever reported for any single-agent molecularly targeted therapy in advanced NSCLC and among all metastatic solid tumors (7). In patients who experienced disease progression after treatment with one or more second-generation ALK-TKIs, lorlatinib demonstrated greater efficacy in those harboring ALK mutations than in those without such mutations (8).

Notably, patients with ALK-positive NSCLC exhibit a high incidence of brain metastases (BMs), with at least 20% presenting with BMs at initial diagnosis (9). In contrast, intraspinal metastasis is an uncommon site of metastatic involvement in NSCLC (10). When intraspinal metastases invade the lumbar spinal canal, they may cause low back pain as well as neurological symptoms and signs in the lower limbs (11). Herein, we report a case of intraspinal metastasis in a patient with ALK-positive lung adenocarcinoma who achieved an overall survival (OS) of 121 months (right-censored, t+), with a duration of treatment (DOT) of 48 months (t+) with lorlatinib as fifth-line therapy.

## 2 Case presentation

**Figure 1:**
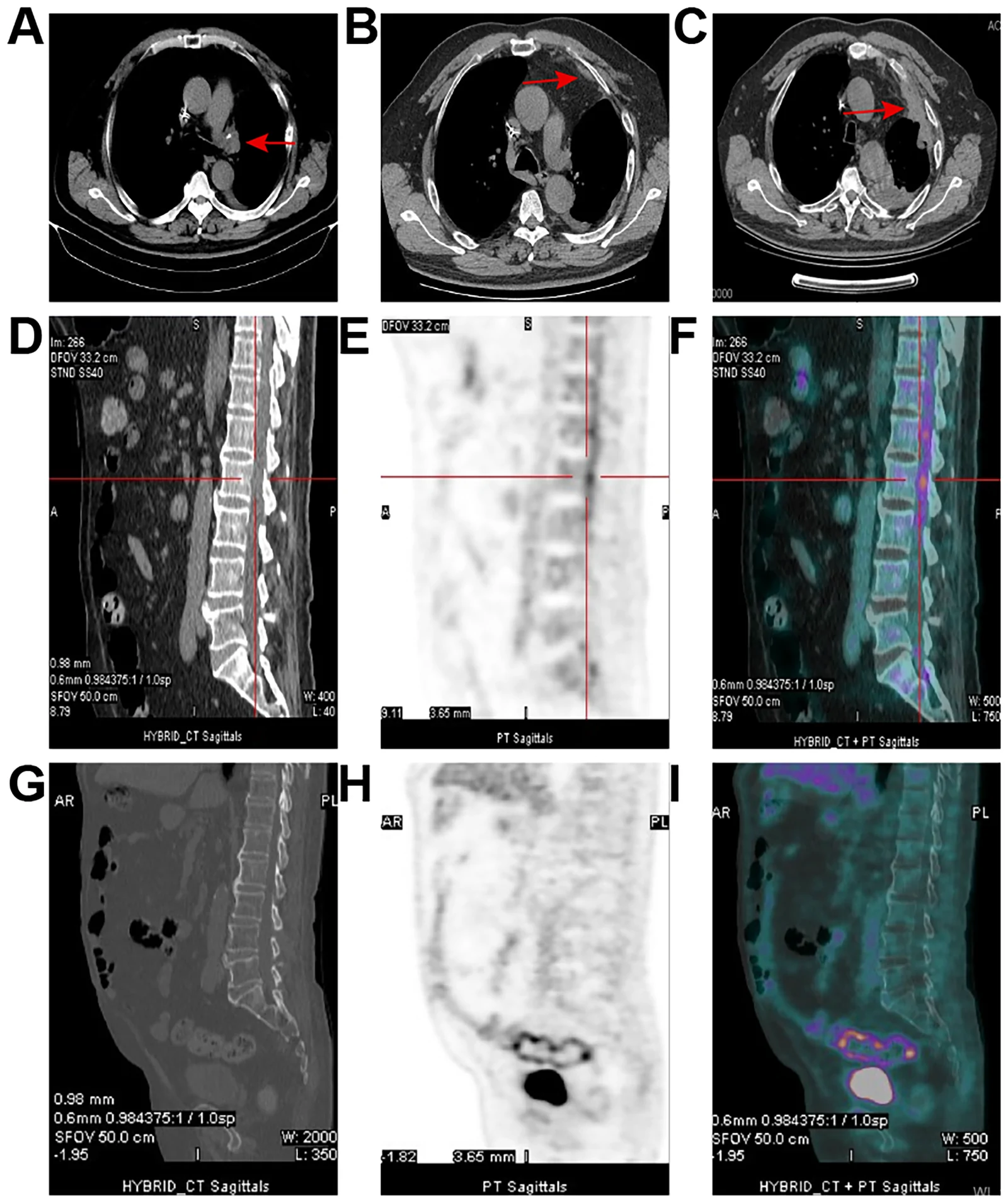
Images of diagnostic imaging results. CT imaging findings **(A-C)**. A preoperative CT imaging on June 8, 2016 revealed a soft-tissue nodule measuring approximately 22 mm × 18 mm **(A)**. A CT scan performed on March 22, 2018, demonstrated bilateral pleural thickening **(B)**. The nodular pleural thickening involving the left hemithorax noted on April 18, 2019 **(C)**. Findings from the PET/CT examination. **(D-F)** PET/CT scan performed on May 26, 2022, revealed segmental hypermetabolism with increased glucose uptake in the thoracic, lumbar, and sacral spinal canals, with a SUVmax of 7.16, raising concern for newly developed metastatic disease. **(G-I)** PET/CT scan performed on October 31, 2025, showed that the previously increased FDG uptake in the thoracolumbosacral spinal canal observed on May 26, 2022, was no longer evident. PET/CT was utilized to evaluate treatment response.

**Figure 2:**
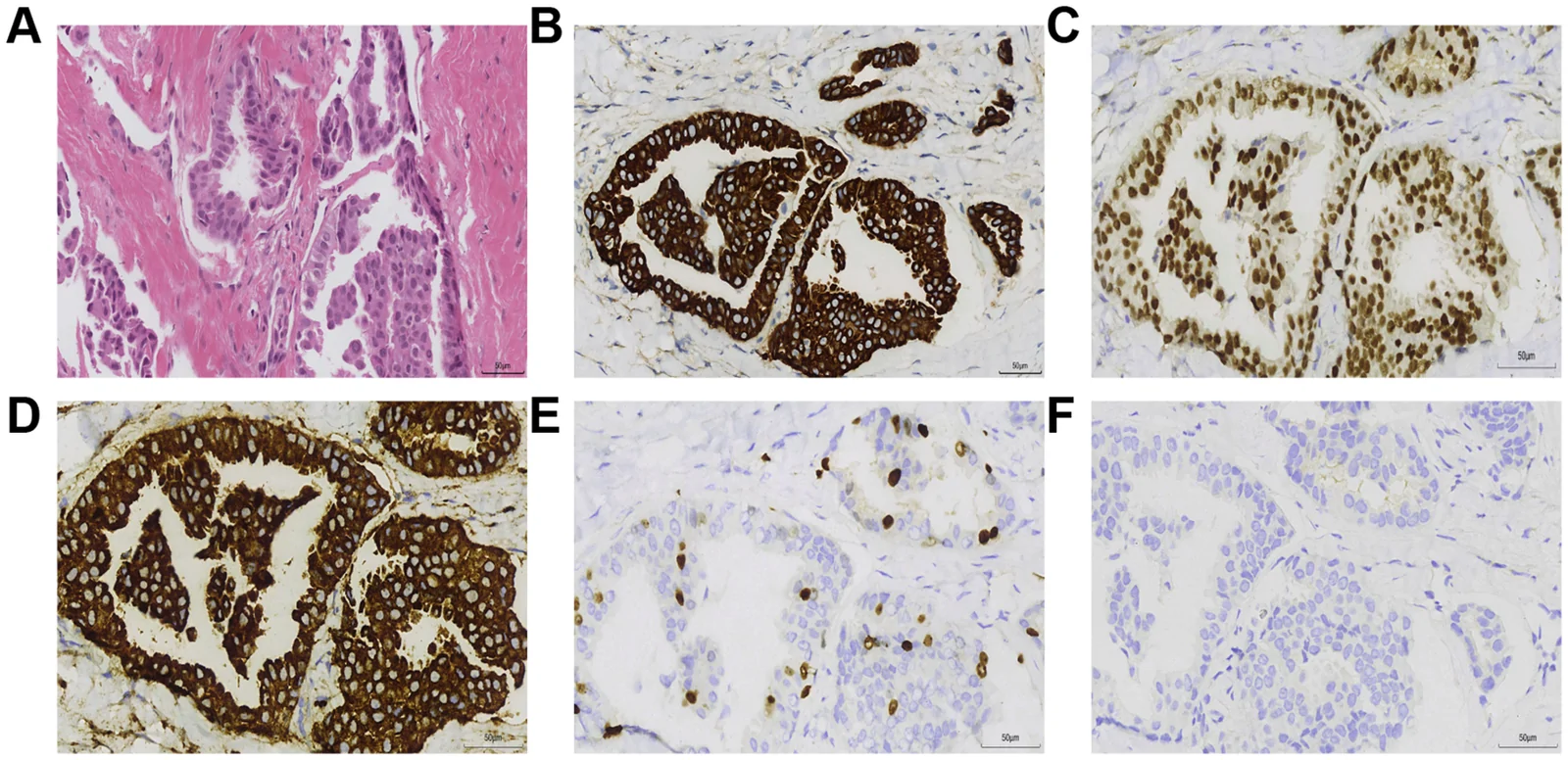
Pathological findings obtained from a needle biopsy performed at the time of the third disease recurrence on December 24, 2019. **(A)** Hematoxylin and eosin (H&E) staining. **(B-F)** Immunohistochemical staining. **(B)** CK7, **(C)** TTF-1, **(D)** NapsinA, **(E)** Ki-67, and **(F)** CDX-2. Scale bar: 50 μm; magnification: ×400.

A 43-year-old male patient presented to our hospital on June 8, 2016. A chest computed tomography (CT) imaging revealed a well-circumscribed soft-tissue nodule measuring approximately 22 mm × 18 mm located in the subpleural region of the left hemithorax (**Figure 1A**). He underwent video-assisted thoracoscopic left upper lobectomy with systematic mediastinal lymph node dissection on June 13, 2016. Postoperative pathological staging was pT2N1M0 according to the 7th edition of the American Joint Committee on Cancer (AJCC) staging system, corresponding to stage IIA pulmonary adenocarcinoma (**Table 1**; **Figure 2**). Postoperative adjuvant chemotherapy consisted of four cycles of intravenous pemetrexed (0.9 g) plus nedaplatin (140 mg), administered from July 12 to September 23, 2016. A CT scan performed on March 22, 2018, demonstrated bilateral pleural thickening (**Figure 1B**). On April 4, 2018, percutaneous biopsy of a left pleural nodule confirmed disease progression, representing the first tumor recurrence, without common driver gene mutations identified (EGFR and ALK) (**Table 1**). Systemic first-line therapy was initiated on April 6, 2018, consisting of five cycles of intravenous pemetrexed (0.8 g, Day 1) plus carboplatin (500 mg, Day 1), administered every three weeks. The treatment cycles were delivered on April 6, April 26, May 17, June 7, and July 7, 2018. On August 22, 2018, cytological examination of pleural effusion confirmed disease persistence (**Table 1**). The patient subsequently received single-agent maintenance chemotherapy with 6 cycles of intravenous pemetrexed (0.8 g, Day 1) every three weeks from August 22, 2018 to January 28, 2019. A chest CT scan performed on April 18, 2019, revealed nodular pleural thickening involving the left hemithorax, accompanied by a left-sided pleural effusion suggestive of progressive disease (PD) (**Figure 1C**). Following the second relapse, second-line intravenous chemotherapy consisting of albumin-bound paclitaxel (300 mg, Day 1), carboplatin (500 mg, Day 1), and bevacizumab (500 mg, Day 2). This regimen was administered on April 24, May 29, July 9, and August 12, 2019. Maintenance therapy was then initiated with three cycles of intravenous pemetrexed (0.8 g), administered between September 12 and November 12, 2019. On December 18, 2019, CT-guided biopsy of a left anterior pleural nodule confirmed disease recurrence, representing the third recurrence (**Table 1**). Following genetic testing that identified an ALK mutation on December 24, 2019 (**Table 1**), third-line targeted therapy with crizotinib capsules (250 mg twice daily) was initiated and continued from December 24, 2019, to March 5, 2021. Palliative radiotherapy targeting metastatic lesions in the left anterior chest wall and ribs was initiated on December 25, 2019, with a total prescribed dose (DT) of 30 Gy delivered in 10 fractions (30 Gy/10 Fx). Due to disease progression, alectinib capsules (600 mg twice daily) were administered as fourth-line targeted therapy from March 6, 2021, to June 23, 2022.

**Table 1:** Pathological results.

Date	Pathology results
June 13, 2016	Frozen report: Wedge-resected lung tissue shows peripheral low-grade pulmonary adenocarcinoma (1.5 cm in diameter), with a focal component (approximately 5%) exhibiting a micropapillary growth pattern and suspected pleural invasion.
June 14, 2016	Routine pathology: Specimen from left upper lobectomy with systematic lymph node dissection. The pulmonary mass in the left upper lobe had been resected and evaluated intraoperatively by frozen section (see frozen report). No residual carcinoma was identified in the remaining lung tissue, and the bronchial resection margin was negative for malignancy. Lymph node examination revealed metastatic carcinoma in the peribronchial lymph nodes (2/8). No metastatic involvement was identified in station 10 (0/1), station 11 (0/1), station 5 (0/2), station 7 (0/1), or station 9 (0/3) lymph nodes.
April 4, 2018	The pathology revealed atypical cell nests within the fibrous tissue of the right pleura (anterior and posterior). In the context of the patient’s clinical history, these findings were considered consistent with pleural metastasis from lung adenocarcinoma.
April 16, 2018	Genetic testing for EGFR exons 18, 19, 20, and 21 revealed no mutations. In addition, testing for the EML4-ALK fusion gene revealed a negative finding.
August 22, 2018	A small number of deeply stained atypical cells were identified in the pleural effusion smear, and the presence of adenocarcinoma cells was highly suspected.
December 20, 2019	Atypical epithelial cell clusters were identified in the left pleural smear, and the presence of adenocarcinoma cells was considered possible.
December 24, 2019	Left pleural nodule: Infiltration of adenocarcinoma was identified in the puncture biopsy of the left pleural nodule (**Figure 1A**). Considering the patient’s medical history and immunohistochemical profile, the findings were consistent with metastatic lung adenocarcinoma. Immunohistochemistry showed CK7 (+) (**Figure 1B**), P40 (−), TTF-1 (+) (**Figure 1C**), Napsin A (+) (**Figure 1D**), P53 (−), Ki-67 (approximately 15%, +) (**Figure 1E**), and CDX-2 (−) (**Figure 1F**).
December 31, 2019	Next-generation sequencing of a percutaneous biopsy specimen obtained from left pleural nodule identified two somatic variants: (1) a frameshift indel in TERT exon 2 (E113Rfs*15; VAF = 1.46%); and (2) an ALK–EML4 fusion with genomic breakpoint at chr2:29448283 (fusion-derived allele frequency = 36.56%).

In May 2022, the patient developed progressive weakness in both lower limbs, eventually resulting in an inability to walk, along with urinary retention. Manual muscle testing (MMT) grades and Eastern Cooperative Oncology Group (ECOG) performance status (PS) scores were presented in **Supplementary Figure 1**. The patient had a history of type 2 diabetes mellitus and hypertension for more than 10 years, and a cardiac pacemaker had been implanted in 2016. There was no family history of malignancy. A lumbar CT scan performed on May 20, 2022, demonstrated degenerative changes affecting the lumbar vertebral bodies; however, no evidence of neoplastic disease was identified. A PET/CT scan performed on May 26, 2022, revealed segmental hypermetabolism with increased glucose uptake in the thoracic, lumbar, and sacral spinal canals, raising concern for newly developed metastatic disease (**Figures 1D–F**). Palliative radiotherapy targeting the intraspinal metastases was administered from May 30 to June 13, 2022, delivering a total dose of 30 Gy in 10 fractions (30 Gy/10 Fx) using 6-MV X-ray irradiation. Fifth-line systemic therapy was initiated on June 19, 2022, comprising oral anlotinib capsules (12 mg once daily, administered on Days 1–14 of each 21-day cycle for 4 cycles) and initial oral lorlatinib tablets (100 mg once daily from June 19 to June 30, 2022; 75 mg from July 1 to September 11, 2022). The maintenance therapy with oral lorlatinib tablets (75 mg once daily) from September 12, 2022 to December 31, 2022, and lorlatinib tablets (100 mg once daily) from January 1, 2023 to the present. The results of serum troponin I measurement, transthoracic echocardiography, and electrocardiography reveal no contraindications to initiating treatment with anlotinib capsules (**Supplementary Table 1**).

**Figure 3:**
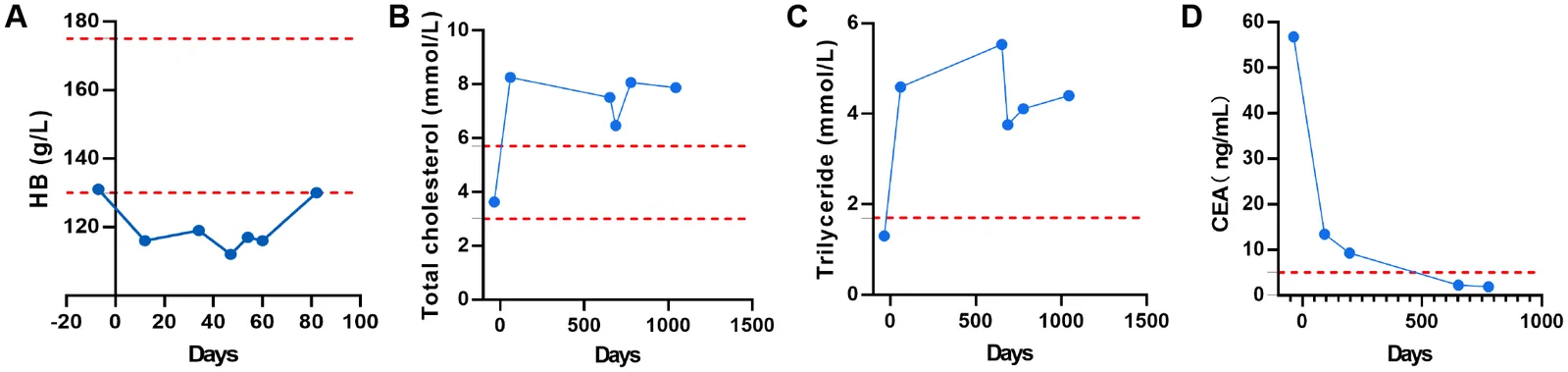
The changes of laboratory test results during the fifth-line treatment, and the timeline of diagnosis and treatment. During fifth-line therapy, the patient exhibited a decline in hemoglobin concentration **(A)**, elevated total cholesterol **(B)** and triglyceride levels **(C)**, and a sustained reduction in carcinoembryonic antigen (CEA) **(D)**. The laboratory test results were plotted on the y-axis and days post–May 25, 2022 (designated as the reference time point, i.e., x = 0) on the x-axis.

A PET/CT scan performed on October 31, 2025, showed that the previously increased FDG uptake in the thoracolumbosacral spinal canal observed on May 26, 2022, was no longer evident (**Figures 1G–I**). During fifth-line therapy, he exhibited a decline in hemoglobin concentration (**Figure 3A**), elevated total cholesterol (**Figure 3B**) and triglyceride levels (**Figure 3C**), and a sustained reduction in carcinoembryonic antigen (CEA) (**Figure 3D**). According to the Common Terminology Criteria for Adverse Events (CTCAE) version 5.0, treatment-related adverse events were reported as follows: (I) Grade 1 anemia was considerd to likely attritutable to anlotinib or disease; (II) Grade 2 hypercholesterolemia and Grade 3 hypertriglyceridemia were considered likely attributable to lorlatinib/anlotinib therapy; (III) Grade 3 visual hallucination: On July 1, 2022, following a psychiatric evaluation that revealed Grade 3 hallucinations, aggressive behavior, and severe cognitive impairment, the adverse event was determined to be attributable to lorlatinib administration. Olanzapine 10 mg daily was initiated concurrently, and the lorlatinib dose was reduced from 100 mg to 75 mg once daily for six months. Subsequently, the lorlatinib dose was escalated back to 100 mg once daily from January 1, 2023, and maintained at this level thereafter.

**Figure 4:**
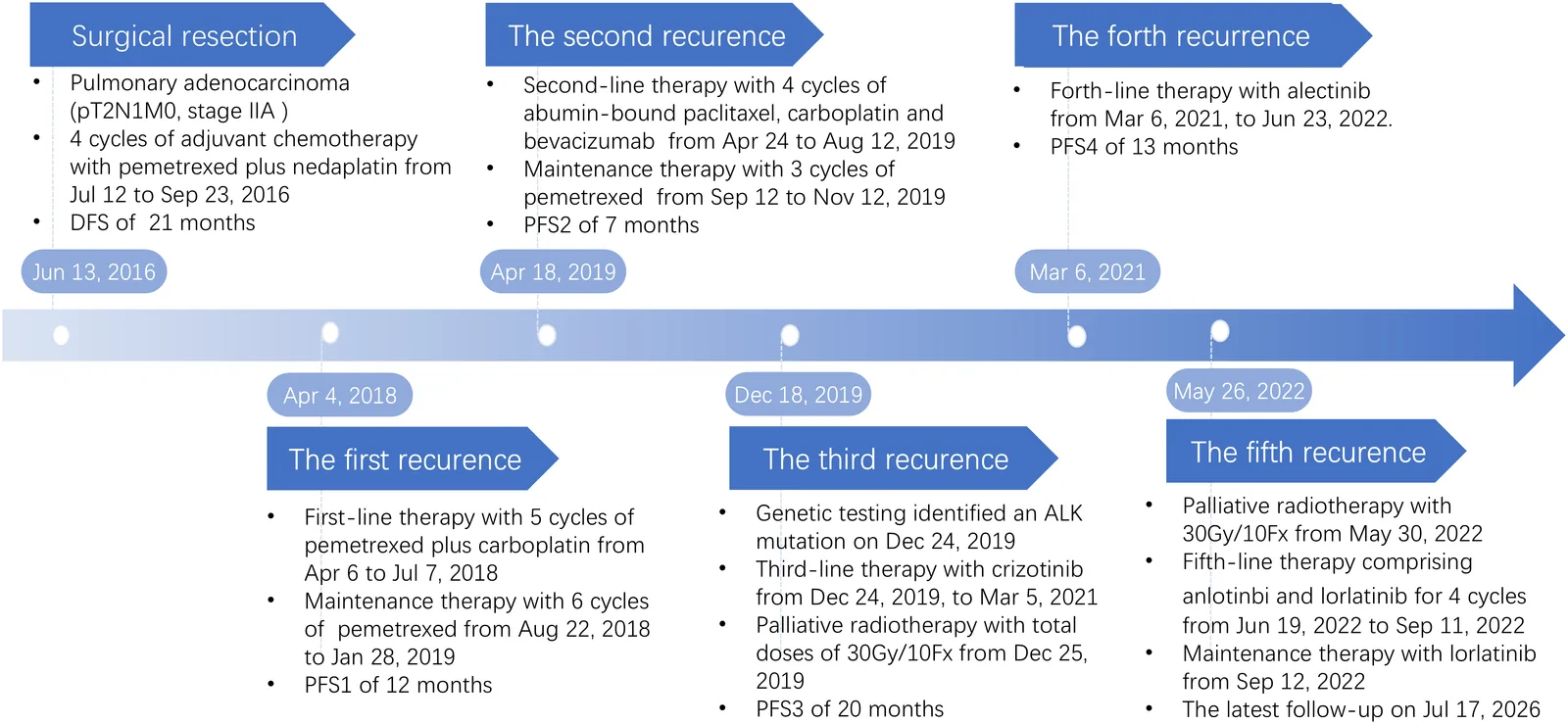
The timeline outlines the chronological sequence of patient diagnosis and treatment interventions.

The most recent comprehensive follow-up visit occurred on December 6, 2025, and the most recent routine follow-up visit was conducted on July 17, 2026. Following a multi-line treatment regimen, the patient achieved an OS duration of 121 months (t+), calculated from the surgical resection performed on June 13, 2016, to the most recent routine follow-up visit on July 17, 2026, and a DOT of 48 months (t+) with lorlatinib administered as fifth-line treatment, spanned from the date of the initial dose (June 19, 2022) to the date of the most recent routine follow-up visit (July 17, 2026). A summary of the patient’s treatment course was presented in **Figure 4**.

## 3 Discussion

In this case, the patient was unable to undergo lumbar MRI due to the presence of a cardiac pacemaker. Additionally, the patient declined lumbar puncture for cerebrospinal fluid (CSF) collection and subsequent cytological or molecular analysis. Lumbar CT revealed no evidence of intraspinal tumor lesions; however, PET/CT identified metastatic disease within the thoracolumbar and sacral spinal canal. Following a multidisciplinary team (MDT) discussion involving specialists from thoracic surgery, medical oncology, radiation oncology, pathology, and diagnostic radiology, intraspinal metastasis was presumed to be leptomeningeal (pial) metastasis (LM), lorlatinib in combination with anlotinib was selected as the fifth-line systemic therapy following palliative radiotherapy to the thoracolumbar and sacral regions. The patient achieved full recovery of urinary function and lower limb muscle strength (MMT grades 5) and restoration of normal ambulation.

LM occurs in 3–5% of NSCLC patients, with higher incidence in those harboring ALK rearrangements (12). However, in a retrospective study, the ALK gene mutation was associated with a decreased risk of meningeal metastasis (13). Patients with NSCLC and LM have a poor prognosis, with a mOS of approximately 3 months with current standard therapies and up to 11 months with emerging novel treatment modalities (12). Lorlatinib enhances blood–brain barrier (BBB) permeability by downregulating secreted phosphoprotein 1, leading to inhibition of vascular endothelial growth factor (VEGF), transforming growth factor-β, and claudin expression, and consequent reduction in tight junction integrity between BBB endothelial cells (14). Lorlatinib shows enhanced efficacy against leptomeningeal metastases. However, its effect is also influenced by CSF-specific factors, including driver/resistance alterations in CSF, unbound active drug fraction, efflux transporter activity, tumor burden, CSF flow dynamics, and compartmental heterogeneity (15). *In vitro* experiments indicated that crizotinib resistance was mediated by angiogenesis through the Janus kinase 2 (JAK2)/signal transducer and activator of transcription 3 (STAT3)–chemokine (C-C motif) ligand 20 (CCL20)–vascular endothelial growth factor A (VEGFA)/(interleukin-6) IL-6 axis (16). Anti-angiogenic therapies may augment antitumor immunity and thereby enhance the therapeutic efficacy of ALK inhibitors, including alectinib, as evidenced by a prospective observational study (17). A phase I trial of the ALK inhibitor crizotinib in combination with the VEGF inhibitor pazopanib in patients with advanced solid malignancies demonstrated moderate clinical activity (18). An *in vivo* study using a xenograft mouse model demonstrated that combination therapy with anti-vascular endothelial growth factor receptor 2 (VEGFR2) antibodies and molecularly targeted agents, such as ALK inhibitors, holds promise as a therapeutic strategy for oncogene-driven NSCLC (19). In the Phase III, randomized, controlled trial of anlotinib (ALTER-0303), patients harboring EGFR or ALK mutations were eligible for enrollment following disease progression on standard targeted therapy (20). Anlotinib capsules, a vascular endothelial growth factor receptor tyrosine kinase inhibitor, have been approved by the National Medical Products Administration (NMPA) of China for the treatment of patients with recurrent or metastatic NSCLC who have received two prior lines of systemic chemotherapy. In patients harboring EGFR or ALK genomic alterations, anlotinib may be considered following completion of guideline-recommended targeted therapy and two lines of systemic chemotherapy.

Several factors likely contributed to the favorable long-term outcome. (I) The patient underwent repeat biopsies after multiple recurrences, and the results guided both local and systemic treatment decisions. Herein, the patient underwent four pathological evaluations, including one postoperative specimen and three biopsies performed after disease recurrence, as well as two additional genetic tests. Molecular diagnostics now play a pivotal role in guiding treatment strategies (21). Re-biopsy is recommended to guide optimal therapeutic selection following disease relapse in patients receiving ALK-TKIs (22). In this case of NSCLC, molecular profiling at initial recurrence identified no actionable driver alterations (i.e., EGFR and ALK wild-type), while repeat biopsy at the third disease recurrence revealed an ALK gene mutation. Among patients with ALK-positive NSCLC who had received at least one prior second-generation ALK-TKI, treatment with lorlatinib yielded a median duration of response (mDOR) of 9.6 months, a mPFS of 6.6 months, and a mOS of 20.7 months (23). The median duration of treatment (DOT) with lorlatinib was 161 days overall, 147 days in the second-line setting and 244 days in the third-line or later setting, according to a real-world study conducted in Japanese patients with ALK-positive NSCLC who received lorlatinib following alectinib therapy (24). (II) Although genetic testing at the first relapse indicated ALK wild-type disease, subsequent testing at the third relapse revealed ALK positivity, indicating the importance of reassessment and the potential need for ALK-TKI therapy during disease evolution. (III) palliative radiotherapy followed by ALK-TKI–based targeted therapy provided an effective strategy for rapid local disease control and prolonged PFS in this ALK-positive patient. The patient underwent two sessions of palliative radiotherapy. At the third recurrence, radiotherapy was directed at metastatic lesions in the left chest wall and ribs, followed by targeted therapy with crizotinib. At the fifth recurrence, radiotherapy was administered to intraspinal lesions, after which the patient received targeted therapy with lorlatinib and anlotinib. Timely palliative radiotherapy can provide rapid symptom relief and facilitate subsequent intensive treatment strategies in patients with metastatic NSCLC presenting with oncologic emergencies (25). Growing evidence indicates that radiotherapy in advanced NSCLC plays not only a palliative role but also an increasingly important therapeutic role (26). Overall, molecularly targeted therapeutic agents have demonstrated synergistic effects with radiotherapy in the treatment of patients with BMs (27).

Admittedly, the administration of fifth-line therapy was associated with several limitations: (I) At the time of diagnosing the fifth recurrence, diagnostic assessment relied exclusively on PET/CT imaging; complementary modalities, including contrast-enhanced MRI and cerebrospinal fluid cytology, were not utilized. MRI was contraindicated in patients with implanted cardiac pacemakers due to safety concerns related to electromagnetic interference and the potential for device malfunction. Lumbar CT revealed no evidence of intraspinal tumor lesions; however, PET/CT identified metastatic disease within the thoracolumbar spinal canal, presumed to be leptomeningeal (pial) metastasis. Additionally, the patient declined lumbar puncture for CSF collection and subsequent cytological or molecular analysis. (II) At the fifth relapse, next-generation sequencing was not performed to elucidate the underlying mechanism of relapse or to identify potential novel driver mutations. (III) Following the fifth recurrence, standardized longitudinal imaging surveillance was not instituted, thereby precluding precise temporal characterization of tumor dynamics.

In conclusion, following the fifth disease recurrence, the patient who diagnosed with ALK–EML4-positive NSCLC and intraspinal metastasis, received palliative radiotherapy, followed by combination therapy with lorlatinib and anlotinib, and subsequently transitioned to lorlatinib monotherapy as maintenance treatment. This sequential therapeutic strategy resulted in a truncated DOT (lorlatinib) of 48 months (t+) and a truncated OS of 121 months (t+). It should be acknowledged that the level of evidence provided by a case report is inherently limited. Nevertheless, given the rarity of intraspinal metastasis, conducting large-scale clinical trials to generate higher-level evidence remains challenging.

## Data Availability

The raw data supporting the conclusions of this article will be made available by the authors, without undue reservation.

## Supplementary material

Supplementary Figure 1 Manual muscle testing (MMT) grades and ECOG performance status scores assessed from the time point prior to the first PET/CT examination through to the time of the final follow-up. May 25, 2022 was designated as the reference time point (Day 0).
