# Supplementary material for: Advanced ALK–EML4 fusion–positive non-small cell lung cancer with intraspinal metastases: a case report
Source: Front Oncol. 2026 Sep 3;16:1916641. doi: 10.3389/fonc.2026.1916641 (PMC13581568; doi:10.3389/fonc.2026.1916641)
Supplement: Supplementary file 1 [file Table1.docx]

Table S1. Baseline assessments prior to initiation of anlotinib-therapy

| Date | Examination items | Results |
| --- | --- | --- |
| April 28, 2022 | Laboratory testing | An elevated serum troponin I level of 0.061 ng/mL (reference range: 0–0.030 ng/mL). |
| May 20, 2022 | Transthoracic echocardiography | A preserved left ventricular ejection fraction (LVEF) of 66%. |
| May 24, 2022 | Electrocardiography | A paced rhythm with a heart rate of 78 beats per minute. |
